# A Young Procedure Under a Mature Standard: The Evolving Evidence for GAE

**DOI:** 10.1007/s00270-026-04564-7

**Published:** 2026-08-07

**Authors:** Federico Collettini, Florian Nima Fleckenstein

**Affiliations:** 1 Department of Radiology, Charité University Medicine Berlin, Charitéplatz 1, Charité Campus Mitte (CCM), 10117 Berlin, Berlin Germany; 2 Berlin Institute of Health (BIH), Anna-Louisa-Karsch-Straße 2, 10178 Berlin, Germany

Few interventional procedures have progressed as rapidly from their initial description to widespread clinical interest as genicular artery embolization (GAE). The considerable enthusiasm surrounding the technique has been reflected not only in its widespread clinical adoption by both academic and non-academic centers, but also in the large body of studies evaluating its safety and effectiveness. Since then, the science of musculoskeletal embolization has rapidly developed along two axes. In breadth, the range of indications has expanded far beyond the knee to include other joints, sports-related injuries, and tendinopathies. In depth, the evidence has progressed from early case series to larger prospective studies and, ultimately, to the first randomized sham-controlled trials. An excellent overview and synthesis of the research conducted to date on GAE is provided by Taheri Amin et al. in the current issue of CVIR [1]. Their systematic review and meta-analysis represent the most comprehensive assessment of GAE to date. By pooling 45 studies involving 2205 patients, the authors substantially expand the existing evidence base while maintaining clear methodological rigor. The pooled improvements in visual analogue scale scores, WOMAC scores, and KOOS all exceed the thresholds for clinical relevance. The safety profile is equally reassuring and is characterized predominantly by transient skin discoloration and self-limiting hematomas. No complications of modified CIRSE grade 3 or higher were reported. What particularly distinguishes this work is its intellectual honesty. The authors apply a formal GRADE assessment, report prediction intervals, and systematically separate overlapping cohorts. The meta-analysis also frames the central tension in the evidence with remarkable clarity. The treatment effect within the intervention groups is substantial and sustained; nevertheless, neither of the two included RCTs demonstrated superiority over sham treatment, and the certainty of the evidence remains low. This discrepancy, which has surprised some observers, is regarded by others as not only plausible but even predictable. It should be understood not merely as an expression of the well-known complexity of sham-controlled trials in osteoarthritis, but also as a snapshot of a young therapy in the midst of its maturation, a phase during which the timing of an RCT may itself be open to debate [2]. Over recent years, advances in our understanding of GAE, angiographic technique, embolic materials, and procedural endpoints have led to substantial technical refinement.

An instructive parallel comes from catheter-based renal denervation for resistant hypertension. In 2014, the first blinded, sham-controlled trial, SYMPLICITY HTN-3, failed to show significant benefit, and the therapy was rapidly abandoned [3]. The neutral result was later attributed less to biological failure than to technical immaturity, including incomplete or poorly targeted ablation, limited operator experience, unstable medication adherence, and heterogeneous patient selection. As the technology and procedural strategy matured, rigorous second-generation sham-controlled trials demonstrated genuine blood-pressure reductions [4]. Both leading systems received regulatory approval in 2023 and were subsequently incorporated into clinical guidelines [5]. The lesson cuts both ways: An early negative trial of a device-based, operator-dependent therapy may reflect technical immaturity and limited operator experience as much as failure of the underlying biological concept. Before abandoning that concept, judgment should therefore await rigorously designed studies using mature technology, standardized protocols, and experienced operators beyond the initial learning curve. Until such results are available, neither uncritical enthusiasm nor premature rejection is justified. What is required instead is a balanced and responsible position, exemplified by this meta-analysis. Taheri and colleagues provide more than a collection of pooled estimates. They show where GAE currently stands, openly identify the remaining gaps in knowledge, and establish a methodological benchmark for evaluating this rapidly evolving field of research. Their work is both valuable and timely.
